# *EGFR*突变可切除的NSCLC围手术期辅助靶向治疗研究进展及问题探讨

**DOI:** 10.3779/j.issn.1009-3419.2024.106.11

**Published:** 2024-05-20

**Authors:** SONG Peng, CUI Yong

**Affiliations:** 100050 北京，首都医科大学附属北京友谊医院胸外科; Department of Thoracic Surgery, Beijing Friendship Hospital, Capital Medical University, Beijing 100050, China

**Keywords:** 肺肿瘤, 围手术期治疗, 新辅助治疗, 辅助治疗, 靶向治疗, Lung neoplasms, Perioperative treatment, Neoadjuvant treatment, Adjuvant treatment, Targeted therapy

## Abstract

肺癌在全球范围内仍是导致癌症死亡的首要原因，非小细胞肺癌（non-small cell lung cancer, NSCLC）是肺癌的主要病理类型，占80%左右。在所有NSCLC患者中，约30%初诊时为可切除的早中期NSCLC。近期表皮生长因子受体-酪氨酸激酶抑制剂（epidermal growth factor receptor-tyrosine kinase inhibitors, EGFR-TKIs）在EGFR突变可切除的NSCLC围手术期辅助靶向治疗中取得重大突破，并被指南推荐应用于临床。本文在总结EGFR突变可切除NSCLC围手术期辅助靶向治疗临床研究进展的基础上，针对临床研究中的关键问题进行探讨。

## Abstract (en)

Lung cancer is still the leading cause of cancer death worldwide. Non-small cell lung cancer (NSCLC) is the main pathological type of lung cancer, accounting for about 80%. Approximately 30% of all patients with NSCLC have resectable early and middle stage disease at the time of diagnosis. Recently, the epidermal growth factor receptor-tyrosine kinase inhibitors (EGFR-TKIs) have made a major breakthrough in the adjuvant targeted therapy of EGFR-mutated resectable NSCLC, and are recommended by the guidelines for clinical use. In this review, we summarize the clinical research progress of perioperative adjuvant targeted therapy for EGFR-mutated resectable NSCLC, and discuss the key issues in the clinical researches.

非小细胞肺癌（non-small cell lung cancer, NSCLC）占肺癌总发病率的80%左右，但初诊的NSCLC患者中仅约30%有手术切除机会，这些患者的中位5年总体生存（overall survival, OS）率为36%-60% ^[1]^。在NSCLC患者中，19号外显子缺失突变和21号外显子L858R突变是表皮生长因子受体（epidermal growth factor receptor, EGFR）基因最常见的突变^[2]^，这些患者对EGFR-酪氨酸激酶抑制剂（EGFR-tyrosine kinase inhibitors, EGFR-TKIs）的治疗敏感^[3]^。多项研究^[4⇓⇓-7]^均显示EGFR-TKIs治疗EGFR突变NSCLC的疗效优于传统的化疗药物，而且可以提高患者的生活质量。EVIDENCE和ADAURA的研究^[8,9]^表明，与安慰剂或辅助化疗相比，EGFR-TKIs术后辅助治疗可为IB-IIIA期EGFR突变的NSCLC患者提供显著的生存获益，并被推荐用于IB-IIIA期EGFR突变可切除NSCLC患者的术后辅助治疗。鉴于靶向治疗在术后辅助治疗中带来的显著生存获益，新辅助EGFR-TKIs靶向治疗越来越多地被探索用于治疗EGFR突变的可切除NSCLC，目前已完成的新辅助靶向治疗临床试验公布结果普遍显示EGFR-TKIs新辅助靶向治疗的病理缓解率偏低，并且未能带来显著的生存获益，因此尚无靶向药物获批用于可切除NSCLC的新辅助治疗；并且现有的靶向治疗临床试验存在术前用药周期数不固定、新辅助靶向用药与手术时间间隔不统一、术后恢复辅助靶向治疗时间不清晰、术后辅助靶向治疗持续时间不明确等问题。本文将对EGFR突变可切除的NSCLC术后辅助治疗和新辅助治疗研究现状，以及在围手术期辅助靶向治疗中存在的上述问题进行综述和讨论。

## 1 EGFR突变可切除的NSCLC术后辅助治疗研究进展

临床上30%-50%的NSCLC是可手术切除的，对于IB-IIIA期患者以围手术期辅助治疗加根治性手术切除是推荐的治疗方法^[10,11]^。然而围手术期辅助化疗与单纯手术相比仅可使5年生存率提高5%^[12]^，不但有明显的不良反应^[13]^，并且复发率仍然很高，25%-75%的IB-IIIA期患者切除的NSCLC在5年内复发，随着疾病分期复发的风险增加（I期：26%-45%；II期：42%-62%；III期：70%-77%）^[14,15]^。在根治性切除的NSCLC患者中，最常报道的远处复发部位包括肺（11%）、脑（8%）、胸膜（8%）和骨（6%），特别是中枢神经系统的远处复发与预后不良和对健康相关生活质量的重大影响有关^[16⇓-18]^。需要更有效的治疗来延长可切除NSCLC患者的无病生存期（disease-free survival, DFS）和OS。针对EGFR突变可切除NSCLC，全球研究者们已进行了大量术后辅助EGFR-TKIs靶向治疗的探索研究（表1）。

**表 1:** 术后辅助靶向治疗临床试验汇总

Clinical trials	Treatment regimens	Length of exposure to EGFR-TKIs	Stage	No. of patients	Primary end-point	Results for primary end-point
SELECT (2019) NCT00567359^[19]^	Erlotinib	24	IA-IIIA	100	2-year DFS	Positive 2 year DFS: 88%; 95%CI: 0.80-0.93; P=0.0047
ADJUVANT (2018) NCT01405079^[20]^	Gefitinib	24	II-IIIA	111	DFS	Positive for DFS HR=0.56; 95%CI: 0.40-0.79; P=0.001 Negative for OS HR=0.9; 95%CI: 0.64-1.43; P=0.823
	Chemotherapy		II-IIIA	111		
IMPACT (2022) UMIN000006252^[21]^	Gefitinib	24	II	42	DFS	Negative HR=0.92; 95%CI: 0.67-1.28; P=0.63
			III	74		
	Chemotherapy		II	44		
			III	72		
EVAN (2018) NCT01683175^[22]^	Erlotinib	24	IIIA	51	2-year DFS	Positive RR=1.82; 95%CI: 1.19-2.78; P=0.0054
	Chemotherapy		IIIA	51		
EVIDENCE (2021) NCT02448797^[8]^	Icotinib	24	II-IIIA	161	DFS	Positive HR=0.36; 95%CI: 0.24-0.55; P<0.0001
	Chemotherapy		II-IIIA	161		
CORIN (2023) NCT02264210^[23]^	Icotinib	12	IB	63	3-year DFS	Positive HR=0.23; 95%CI: 0.07-0.81; P=0.013
	Placebo		IB	65		
Neal et al. (2021) NCT01746251^[24]^	Afatinib	3	I-III	23	2-year RFS	Negative P=0.55
	Afatinib	24	I-III	22		
ICOMPARE (2023) NCT01929200^[25]^	Icotinib	12	II-IIIA	55	DFS	Positive HR=0.51; 95%CI: 0.28-0.94; P=0.029
	Icotinib	24	II-IIIA	54		
ADAURA (2023) NCT02511106^[9,26]^	Osimertinib	36	IB	107	DFS	Positive HR=0.17; 95%CI: 0.11-0.26; P<0.001
			II	115		
			IIIA	117		
	Placebo		IB	109		
			II	116		
			IIIA	118		

EGFR-TKIs: epidermal growth factor receptor-tyrosine kinase inhibitors; DFS: disease-free survival; RFS: recurrence-free survival; HR: hazard ratio; CI: confidence interval.

SELECT研究^[19]^是一项单臂、多中心、II期临床研究，旨在评估厄洛替尼用于IA-IIIA期EGFR突变型NSCLC术后辅助治疗的疗效和安全性，结果表明术后辅助厄洛替尼治疗的患者DFS显著优于历史对照组，2年DFS率达到了88%。ADJUVANT研究^[20]^是一项旨在比较术后辅助靶向治疗与辅助化疗的随机III期临床研究，结果显示吉非替尼组中位DFS较化疗组显著延长（P=0.0054），疾病复发风险下降40%；两组中位OS没有统计学差异（75.5 vs 62.8个月，P=0.674），辅助靶向治疗的DFS获益并未转化为显著的OS获益，但吉非替尼组相较于化疗组OS延长了近13个月。IMPACT研究^[21]^是一项随机III期临床研究，旨在探索吉非替尼作为II-III期EGFR突变NSCLC患者辅助治疗的疗效。该研究与ADJUVANT研究在研究设计方面非常相似，但却得到了不同的研究结论。IMPACT研究结果显示靶向治疗组与化疗组的中位DFS相比较未观察到统计学差异（35.9 vs 25.1个月，P=0.63），5年OS率分别为78.0%和74.6%，同样未观察到统计学差异[危险比（hazard ratio, HR）=1.03，P=0.89]。EVAN研究^[22]^是一项多中心、随机II期临床研究，旨在对比术后辅助厄洛替尼和化疗治疗IIIA期EGFR突变NSCLC患者的疗效，研究结果表明靶向治疗组较化疗组的中位DFS延长21.4个月，2年DFS率提升36.7%；中位OS延长近2年，而5年生存率直接提升了33%。EVIDENCE研究^[8]^是一项III期临床研究，旨在比较术后辅助埃克替尼和化疗治疗EGFR突变II-IIIA期NSCLC患者的疗效，研究结果显示，埃克替尼组中位DFS较化疗组延长约2年（HR=0.36, P<0.0001），3年DFS率提高31.41%（63.88% vs 32.47%）。CORIN是一项随机II期临床试验（NCT02264210）^[23]^，入组了行根治性切除的IB期EGFR突变的NSCLC患者，以1:1随机接受辅助埃克替尼12个月或观察，直到疾病复发。结果显示埃克替尼组的3年DFS率明显高于观察组（96.1% vs 84.0%, P=0.041），埃克替尼组对比观察组的DFS亦明显延长且复发或死亡风险降低77%。CORIN研究确证了埃克替尼辅助治疗能够显著提高IB期EGFR突变NSCLC患者术后的3年DFS，且具有良好的安全性与耐受性，为IB期EGFR突变NSCLC患者提供了全新的辅助治疗选择。Neal等^[24]^开展了一项行根治性手术切除的I-III期EGFR突变NSCLC患者术后辅助阿法替尼治疗3个月对比治疗2年的随机II期临床研究（NCT01746251），结果表明2年治疗组的中位无复发生存期（recurrence-free survival, RFS）较3个月治疗组延长15.8个月（58.6 vs 42.8个月），术后辅助阿法替尼治疗2年比治疗3个月仅可降低11%的2年复发率（81% vs 70%）。ICOMPARE研究^[25]^是一项多中心、II期随机对照的术后辅助治疗研究，旨在比较不同治疗时长的埃克替尼（2 vs 1年）对完全切除后的II-IIIA期EGFR敏感突变肺腺癌患者的疗效和安全性，结果显示2年组患者中位DFS显著高于1年组（48.9 vs 32.9个月，P=0.029），2年组患者中位OS为75.8个月，而1年组无法评价（HR=0.34, P=0.0317）；与1年组相比，2年组术后辅助埃克替尼治疗显著改善了II-IIIA期EGFR敏感突变肺腺癌患者的DFS并提供了OS获益。ADAURA研究^[9]^是首个第三代EGFR-TKIs术后辅助治疗IB-IIIA期EGFR突变NSCLC的III期临床研究，不论在IB-IIIA期人群还是在II-IIIA期人群中，奥希替尼均可使患者疾病复发风险相对下降70%以上。相比于安慰剂组，奥希替尼治疗使患者的5年生存率绝对值提升近10%，死亡风险相对下降51%（HR=0.49, P<0.0001），实现了有统计学差异和临床意义的OS改善^[26]^。基于该临床试验，奥希替尼成为首个被推荐用于术后辅助治疗IB-IIIA期EGFR突变NSCLC患者。

上述研究表明，术后辅助EGFR-TKIs治疗比术后辅助化疗或安慰剂显著降低了IB-IIIA期EGFR突变NSCLC患者的疾病进展或死亡风险，延长了患者的RFS和长期生存，但总体来看辅助第一代EGFR-TKIs治疗在延长患者的DFS和OS方面亚于第三代EGFR-TKIs。第一代EGFR-TKIs在改善患者预后方面弱于第三代EGFR-TKIs的主要原因可能是使用第一代EGFR-TKIs引起获得性耐药的发生时间短于第三代EGFR-TKIs。EGFR-TKIs辅助治疗发生获得性耐药的机制包括EGFR依赖性机制，主要是获得T790M突变、C797S突变等，这些突变阻碍了靶向药物的结合，其治疗策略有赖于第三、四代EGFR-TKIs药物；以及EGFR非依赖性机制，包括间质上皮细胞转化因子（mesenchymal epithelial transition factor, MET）/人表皮生长因子受体2（human epidermal growth factor receptor 2, HER2）扩增、Kirsten大鼠肉瘤病毒癌基因（kirsten rat sarcoma viral oncogene homolog, KRAS）/v-raf鼠肉瘤病毒癌基因同源体B1（v-raf murine sarcoma viral oncogene homolog B1, BRAF）/磷脂酰肌醇-4,5-二磷酸3-激酶催化亚基α（phosphatidylinositol-4,5-bisphosphate 3-kinase catalytic subunit alpha, PIK3CA）突变、致癌融合和组织学转化等，其治疗策略需要联用相应靶点的靶向药物或改用化疗/免疫治疗^[27]^。上述各种耐药的机制已经被广泛研究，但迄今为止还没有针对每种类型的EGFR-TKIs耐药的全面和标准化的解决方案。通过下一代测序（next-generation sequencing, NGS）技术对血液循环肿瘤DNA（circulating tumor DNA, ctDNA）或组织样本的耐药机制进行检测并明确耐药机制，对于针对不同耐药机制制定个性化治疗策略具有重要意义。

## 2 EGFR突变可切除的NSCLC新辅助治疗研究进展

为减少可切除NSCLC的微转移灶、提升R0切除率和生存率，术前新辅助治疗模式的探索成为近年来的热点。可切除NSCLC进行新辅助治疗具有多种潜在益处^[28]^，包括新辅助治疗比辅助治疗耐受性更好、早期全身治疗可控制微转移灶，以及新辅助治疗后患者需要的手术切除范围可能较前减小，R0切除率提高。新辅助治疗允许对生存评估（OS、DFS）进行替代终点评估，如治疗反应的临床、病理或相关生物标志物评估。术前新辅助治疗的依从性高于术后辅助治疗，也有助于评估体内治疗效果，并可能指导辅助治疗。一项针对可切除NSCLC患者新辅助治疗试验的综述^[29]^显示，与单纯手术相比新辅助化疗的5年生存率提高了5%，相对死亡风险降低了13%（HR=0.87）。因此，新辅助化疗被指南推荐用于EGFR突变可切除IB-IIIA（N2）期NSCLC术前治疗，但由于生存获益有限，研究者们正在积极探索新辅助靶向治疗EGFR突变可切除IB-IIIA（N2）期NSCLC患者的安全性、有效性及临床获益（表2）。

**表 2:** 新辅助靶向治疗临床试验汇总

Clinical trials	Drugs	Length of exposure to EGFR-TKIs	No. of patients	Stage	ORR (%)	PFS/EFS/DFS (mon)	OS (mon)	Pathologic downstaging (%)	Pathologic remission (%)	^R0 resection ^(%)
NCT00188617 (2009)^[30]^	Gefitinib	28 days	36	I	11.0	-	-	43.0	-	-
NCT01833572 (2021)^[31]^	Gefitinib	42 days	33	II-IIIA	54.5	DFS: 33.5	-	20.0	MPR: 24.2; pCR: 12.1	87.9
NCT01217619 (2020)^[32]^	Erlotinib	4-7 weeks	15	IIIA	67.0	PFS: 12.1; DFS: 10.2	51.0	-	MPR: 67.0; pCR: 0.0	-
NCT01407822 (2019)^[33]^	Erlotinib	42 days	37	IIIA-N2	-	PFS: 21.5	42.2	10.8	MPR: 9.7; pCR: 0.0	73
NCT00600587 (2015)^[34]^	Erlotinib	42 days	12	IIIA-N2	58.3	PFS: 6.9	14.5	16.7	-	25
Chen et al. (2018)^[35]^	Erlotinib	9 weeks	43	IIIA	67.4	-	56	-	-	79.10
NCT04201756 (2023)^[36]^	Afatinib	1-9 cycles	47	III	70.2	-	-	57.6	MPR: 9.1; pCR: 3.0	87.9
NCT03433469 (2023)^[37]^	Osimertinib	1-2 cycles	27	I-IIIA	48.0	DFS: 32.0	-	44.0	MPR: 15.0; pCR: 0.0	-
ChiCTR1800016948 (2023)^[38]^	Osimertinib	6 weeks	38	IIA-IIIB-N2	71.0	-	-	46.9	MPR: 10.7; pCR: 3.6	93.8

ORR: objective response rate; EFS: event-free survival; PFS: progression-free survival; OS: overall survival; MPR: major pathologic response; pCR: pathologic complete response.

一项开放标签、单臂II期研究（NCT00188617）^[30]^报告，吉非替尼用于I期NSCLC患者的新辅助治疗是一种安全可行的治疗方案，客观缓解率（objective response rate, ORR）为11%，EGFR突变是预测治疗反应的最佳生物标志物。另一项单臂II期研究（NCT01833572）^[31]^表明，吉非替尼新辅助治疗EGFR突变II-IIIA期NSCLC患者的主要病理缓解（major pathologic response, MPR）率为24.2%，ORR为54.5%。在一项针对中国IIIA期NSCLC（NCT01217619）患者的研究^[32]^中，厄洛替尼与化疗相比是一种有效的新辅助治疗方案，厄洛替尼在ORR（67% vs 19%）、MPR率（67% vs 38%）和OS（51.0 vs 20.9个月）方面高于基于顺铂的双药化疗。CTONG1103（NCT01407822）是一项旨在比较新辅助厄洛替尼与化疗在IIIA-N2期EGFR突变NSCLC患者中疗效的II期临床研究^[33]^，研究发现厄洛替尼组和化疗组的ORR分别为54.1%和34.3%。化疗组没有患者取得MPR或病理完全缓解（pathologic complete response, pCR），厄洛替尼治疗组MPR率仅为9.7%，pCR率也为0%。厄洛替尼组中位PFS比化疗组延长10.1个月，然而延长的PFS并不能转化为OS的显著获益。2015年公布的厄洛替尼新辅助临床研究（NCT00600587）^[34]^共纳入了24例IIIA-N2期的NSCLC患者，随机分配到厄洛替尼或吉西他滨/卡铂组，研究结果表明厄洛替尼相比于化疗可提高患者的新辅助治疗的ORR（58.3% vs 25.0%），但并没有给患者带来PFS（6.9 vs 9.0个月）或OS（14.5 vs 28.1个月）获益。在国内一项厄洛替尼对比化疗新辅助治疗IIIA期EGFR突变肺腺癌的随机对照研究^[35]^中，新辅助靶向治疗组与新辅助化疗组相比，ORR（67.4% vs 44.2%）和组织学治疗有效率（65.1% vs 41.9%）均显著提高。TEAM-LungMate 004研究（NCT04201756）^[36]^是一项旨在评估阿法替尼新辅助治疗EGFR突变阳性III期NSCLC可行性的II期临床研究，结果表明接受阿法替尼新辅助治疗患者的ORR为70.2%；R0切除率为87.9%，MPR率为9.1%，pCR率为3.0%。一项奥希替尼新辅助治疗I-IIIA期EGFR突变NSCLC的临床试验（NCT03433469）^[37]^结果表明，奥希替尼新辅助治疗后患者MPR率为15%，pCR率为0%，ORR为52%，术后中位DFS为32个月。NEOS研究（ChiCTR1800016948）^[38]^是探索奥希替尼新辅助治疗EGFR突变可切除IIA-IIIB期NSCLC患者疗效的II期临床研究，ORR为71.1%，MPR率为10.7%，pCR率为3.6%，R0手术切除率为93.8%。NEOS研究中奥希替尼新辅助治疗的ORR高达71.1%，优于第一代EGFR-TKIs新辅助治疗的结果。

与新辅助化疗联合免疫治疗相比，EGFR-TKIs单药新辅助靶向治疗的MPR或pCR率仍然较低。一方面由于EGFR靶向药不同于传统的化疗药物，其主要作用是抑制肿瘤细胞增殖并促进肿瘤细胞凋亡，而不是像化疗通过细胞毒性直接杀死癌细胞，化疗同时也参与机体免疫反应的调节，可以调节免疫细胞数量并抑制肿瘤的免疫逃逸，进一步增强免疫治疗的疗效^[39]^；另一方面，现有的新辅助靶向治疗临床试验探索仍处于早期阶段，最佳治疗持续时间尚不清楚，延长新辅助靶向治疗的持续时间也许能够改善MPR或pCR率。正在进行的大型全球多中心奥希替尼新辅助III期临床试验NeoADAURA研究^[40]^将新辅助EGFR-TKIs的治疗时间延长至9周以期取得更好的临床获益，该研究结果值得期待，或可正式开启可切除EGFR突变NSCLC患者新辅助靶向治疗新纪元。

## 3 围手术期辅助EGFR-TKIs靶向治疗方案问题探讨

尽管新辅助治疗时间很短，但目前新辅助治疗时间未明确规定。既往临床研究中第一代EGFR-TKIs新辅助治疗多为6或8周，如CTONG1103研究^[33]^和NCT00600587研究^[34]^纳入了IIIA-N2期肺癌患者术前接受厄洛替尼新辅助治疗6周，主要研究终点ORR分别为54.1%和58.3%。NCT01217619研究^[32]^中IIIA-N2期NSCLC患者则接受厄洛替尼新辅助治疗8周，ORR为42.1%。2018年Chen等^[35]^发表关于厄洛替尼对比化疗新辅助治疗研究结果，研究纳入86例IIIA期NSCLC患者，接受厄洛替尼新辅助治疗9周，主要研究终点ORR为67.4%。NEOS研究^[38]^中第三代EGFR-TKIs奥希替尼新辅助靶向治疗6周，ORR为71.1%。从上述临床研究中似乎可以看出随着新辅助靶向治疗时间的延长及靶向治疗药物的迭代，新辅助治疗的有效率会进一步提升。因此，随后开展的新辅助EGFR-TKIs靶向治疗临床试验大都把新辅助靶向治疗时间延长至8周以上。NeoADAURA研究中规定新辅助治疗时长为9周，伏美替尼FORESEE研究（NCT05430802）和泽拉替尼新辅助治疗（NCT05469022）EGFR突变IIB-IIIB期NSCLC受试者，同样采用EGFR-TKIs新辅助治疗9周。正在招募的NCT04685070研究中，规定阿美替尼新辅助治疗时长8-16周。综上，新辅助靶向治疗时长仍没有定论，即使同一个研究药物，不同的新辅助研究也会采用不用的治疗时间，新辅助靶向治疗的时长是否对治疗结局产生影响，仍需要后续开展相关研究进行验证。尽管新的辅助靶向治疗会延长从诊断到手术的时长，可能会导致患者疾病进展的风险增加，但从现有的临床试验来看使用第三代EGFR-TKIs新辅助靶向治疗可将ORR提高到70%以上，并且随着新辅助靶向治疗时间的延长，ORR可能会进一步提高。因此我们推荐EGFR突变可切除的NSCLC新辅助治疗选用第三代EGFR-TKIs，并且治疗时间维持9周或以上。

关于手术之前是否需要停用新辅助靶向药，似乎也没有定论。厄洛替尼NCT01217619研究^[32]^中明确规定同意手术者指定新辅助方案后0-2周内接受R0切除，而另一项新辅助研究（NCT00600587）^[34]^方案中规定疾病进展或达到部分缓解的患者，需在停药1周内进行手术。还有更长者，Chen等^[35]^关于厄洛替尼对比化疗的新辅助研究中，提出新辅助治疗结束后3周进行手术。早在2011年，Rizvi等^[41]^发表的吉非替尼与敏感突变表型研究中提出，吉非替尼新辅助用药至手术前2天。奥希替尼NEOS研究^[38]^中提出，靶向新辅助治疗与手术需间隔3-14 d。2021年一项回顾性的奥希替尼新辅助研究^[42]^中，真实世界中奥希替尼新辅助治疗可至手术切除前1天，这是迄今了解到的关于新辅助治疗与手术间隔最短的时间。目前尚无研究新辅助靶向治疗临床研究探讨这一问题，但大部分临床试验设计均规定术前停用一段时间的靶向药，可能是基于以下两个方面考虑：（1）靶向药与麻醉和术后用药之间是否存在潜在的药物相互作用；（2）不停用靶向药是否会增加手术操作难度和术后并发症发生率。

关于术后开始辅助靶向治疗的时间，大多数已发表的术后辅助研究均未触及到这个问题。新辅助和围手术期靶向治疗相关研究并没有统一规定术后开始靶向治疗的时间，ADAURA研究^[9]^要求术后10周内开始靶向治疗；ADJUVANT研究^[20]^要求术后3-6周内开始；IMPACT研究^[21]^规定术后6-10周内开始；CTONG1103研究^[33]^规定术后8周内开始。基于上述临床研究，对于术后辅助靶向而言，一般要求术后10周内开始。目前术后靶向治疗的时间窗问题似乎也从化疗证据延伸，其具体的循证医学证据似乎无从考究，尚无比较性的临床研究探索术后辅助靶向治疗的开始时间不同是否会对疗效产生影响。由于靶向药物的安全性和不良反应耐受性较好，加之术后较早地恢复靶向治疗可以减少围手术期靶向治疗的空窗期，理论上可以更好地控制微转移灶，降低患者的术后复发风险，有可能进一步改善靶向治疗的疗效。因此国内有专家在临床实践过程中，建议患者术后出院便恢复靶向治疗。综上，术后辅助靶向治疗一般在10周内开始，但开始时间的进一步提前需要临床研究提供证据支持。

不同的临床试验规定了不同的术后辅助靶向治疗持续时间，时间1-3年不等。埃克替尼ICOMPARE研究^[25]^对术后辅助靶向治疗持续时间进行了探索，结果显示，2年埃克替尼辅助治疗完全切除后的EGFR突变II-IIIA期肺腺癌患者相比于1年辅助治疗显著延长了DFS（48.9 vs 32.9个月，P=0.029）。埃克替尼2年辅助治疗与更长的OS相关（HR=0.34, P=0.0317），5年OS率显著提高（88% vs 72%, P=0.032），表明术后更长时间的靶向治疗带来DFS获益可能会转化为OS获益。基于该临床试验，EGFR-TKIs术后辅助埃克替尼治疗至少需要2年。随后ADAURA研究^[9]^探索了为期3年术后辅助奥希替尼治疗的疗效，研究结果证实了3年靶向奥希替尼辅助治疗可以为IB-IIIA期NSCLC患者带来DFS巨大获益，并可转化为显著OS获益，因此奥希替尼在国内外均获批用于EGFR敏感突变阳性NSCLC的术后辅助治疗。虽然术后辅助靶向治疗持续时间仍存在争议，但基于上述临床研究目前推荐术后辅助靶向治疗持续时间为2或3年。

综上所述，对于EGFR突变可切除的NSCLC患者的围手术期靶向治疗，术后EGFR-TKIs辅助治疗可以带来显著临床获益，并被指南推荐应用于术后辅助治疗；EGFR-TKIs的新辅助治疗可显著减少肿瘤体积，改善影像学反应，提高根治性手术切除率，但疾病降期或病理缓解的转化率偏低；并且EGFR-TKIs新辅助靶向治疗还存在如下争议：（1）新辅助靶向治疗联合化疗在治疗EGFR突变的可切除NSCLC方面是否优于新辅助靶向治疗；（2）ctDNA动态监测能否准确指导新辅助靶向治疗；（3）并发突变是否对新辅助EGFR-TKIs治疗的反应有影响等问题。未来ANSWER、FORSEE、FRONT和NeoADAURA等临床研究结果有望解决上述争议，并指导EGFR突变的可切除NSCLC的新辅助靶向治疗策略。
